# Multiple drivers behind mislabeling of fish from artisanal fisheries in La Paz, Mexico

**DOI:** 10.7717/peerj.10750

**Published:** 2021-01-29

**Authors:** Adrian Munguia-Vega, Amy Hudson Weaver, José F. Domínguez-Contreras, Hoyt Peckham

**Affiliations:** 1 Conservation Genetics Laboratory and Desert Laboratory on Tumamoc Hill, The University of Arizona, Tucson, AZ, USA; 2 @ Lab Applied Genomics, La Paz, Baja California Sur, Mexico; 3 Sociedad de Historia Natural Niparajá A.C., La Paz, Baja California Sur, Mexico; 4 Instituto Politecnico Nacional-CICIMAR, La Paz, Baja California Sur, Mexico; 5 Ocean Outcomes, Hoyo de Manzanares, Madrid, Spain; 6 Center for Ocean Solutions, Stanford University, Pacific Grove, CA, USA

**Keywords:** Seafood mislabeling, Species substitution, Small-scale fisheries, Seafood chain, Traceability, DNA barcoding

## Abstract

Seafood mislabeling has the potential to mask changes in the supply of species due to overfishing, while also preventing consumers from making informed choices about the origin, quality and sustainability of their food. Thus, there is a need to understand mislabeling and analyze the potential causes behind it to propose solutions. We conducted a COI DNA barcoding study in La Paz, Baja California Sur, Mexico, with 74 samples from fish markets and 50 samples from restaurants. We identified 38 species sold under 19 commercial names, from which at least ∼80% came from local small-scale fisheries. Overall, 49 samples, representing 40% (95% CI [31.4–48.3]) were considered mislabeled in our samples. Based on analyses where species were assigned to three price categories, economic incentives were associated with approximately half of the mislabeling events observed, suggesting that other motivating factors might simultaneously be at play. Using a network approach to describe both mislabeling (when species are mislabeled as the focal species) and substitution (when the focal species is used as substitute for others), we calculated proxies for the net availability of each species in the market. We found that local fish landings were a significant predictor of the net availability of the 10 most important commercial species at retail, but this true availability was masked to the eyes of the final consumer by both mislabeling and substitution. We hypothesize that the level of supply of each species could help explain mislabeling and substitution rates, where species in low supply and high demand could show higher mislabeling rates and rarely be used as substitutes, while species in high supply and low demand could be used as substitutes for the preferred species. Other factors affecting mislabeling include national regulations that restrict the fishing or commercialization of certain species and local and global campaigns that discourage specific patterns of consumption. We discuss how these factors might influence mislabeling and propose some solutions related to communication and education efforts to this local and global challenge.

## Introduction

Seafood mislabeling includes the misrepresentation of the species, origin or weight of a commercial product. Among these, the substitution of one species for another, for example, based on the marketed commercial name and the species identification associated with a genetic barcode, is one of the most important forms of mislabeling. When mislabeling involves a species that is illegally marketed with the intention of deceiving consumers, usually for financial gain, it is recognized as food fraud (Reily, 2018). A number of scientific and popular media reports have documented that seafood mislabeling is a widespread problem in national and international markets (Pardo, Jimenez & Perez-Villarreal, 2016; Warner et al., 2016), including Mexico (Sarmiento-Camacho & Valdez-Moreno, 2018; OCEANA, 2019). Besides economically defrauding consumers (Underwood, 2018), seafood mislabeling has multiple consequences, including indirectly diluting the signals of over-exploitation of species throughout the commercial chain (Crona et al., 2016). Mislabeling also undermines sustainability efforts and compromises various ethical aspects of the seafood trade related to illegal, unregulated and under-reported (IUU) fishing (Marin et al., 2018).

Several studies have related patterns of seafood mislabeling with intentional fraud to increase profits, avoid taxation, conceal illegal species or satisfy market demand (Feitosa et al., 2018; Fox et al., 2018; Reily, 2018; Calosso et al., 2020). Other studies have recently highlighted that mislabeling could also be unintentional due to a lack of effective protocols for identifying, tracing and labeling seafood, or simple mistakes along the commercial chain (Underwood, 2018). Although the causes of mislabeling appear to be diverse and context dependent (Staffen et al., 2017; Donlan & Luque, 2019), there is controversy about what drives seafood mislabeling beyond the common explanation of economic gain.

Standard economic theory explains the relationship between supply and demand of a resource in relation to different price equilibriums. This theory is often used to describe the behavior of many economic activities, including seafood mislabeling. In terms of demand, certain species of fish may have higher demand because of their quality or their traditional identity that has been passed on between generations (Miller, Clarke & Mariani, 2012), or because they became recently popular. The increase in demand of one species could lead to an increase in price, but if its supply is limited, this will also increase the demand of another species that are considered a “substitute good” to the first species. In fact, some studies have suggested that seafood mislabeling could be driven by the need for a constant supply in the market after declines in fisheries landings of preferred species (Jacquet & Pauly, 2008; Miller, Clarke & Mariani, 2012; Crona et al., 2016; Calosso et al., 2020). This would increase the demand (and potentially the fishing pressure) for other species to be used as substitutes for the preferred species. In this way, species with low supply and high demand would show higher mislabeling rates.

Species vary widely in their supply levels due to different factors. Certain species could be available in high quantities throughout the year, either naturally, or due to a mix of capture fisheries, aquaculture and supply from imports. For example, tuna is likely the species with the largest supply in Mexico, due to a combination of industrial fisheries (157,118 tons of tuna *Thunnus* sp. landed in 2015), aquaculture production (7,855 tons produced in 2015) and imports (116,893 additional tons in 2015, while 150,506 tons were exported) (CONAPESCA, 2015). However, other species might show variable levels of supply. Many groupers and snappers show a strong seasonality of their spawning aggregations that make them available to capture fisheries only over a few months during the year (Erisman et al., 2010). In addition, other species of marine fishes may not be imported from elsewhere, their production in aquaculture could be limited or absent, or they could show strong inter- and intra-annual variability in their landings due to environmental or social factors (Pellowe & Leslie, 2017). Additionally, some species traditionally consumed might be in low supply due to overfishing (Marko et al., 2004), for example, long-lived species at high trophic levels, including large fishes that are carnivores and piscivores (Sala et al., 2004; Saenz-Arroyo et al., 2005).

While many of the seafood mislabeling studies have focused on industrial fisheries mainly in developed countries (Pardo, Jimenez & Perez-Villarreal, 2016; Barendse et al., 2019; Luque & Donlan, 2019), the species, patterns and drivers of mislabeling in artisanal fisheries from developing countries may differ due to the unique characteristics of small-scale fisheries. For example, small-scale fisheries could be less determined by global economic factors than is the case with industrial operators, and more influenced by social or cultural values (Smith & Basurto, 2019). In addition, they usually target a larger number of species that are local (Moreno-Báez et al., 2012). High species diversity could increase traceability challenges and contribute to larger mislabeling rates (Carvalho et al., 2017). Furthermore, the implications of mislabeling for small-scale fisheries actors may be particularly important. Small-scale fisheries often play a key role in economic and food security in developing countries, and the capacity of resource users to diversify their target species can be essential to deal with the high variability in fisheries landings (Finkbeiner, 2015).

**Figure 1:**
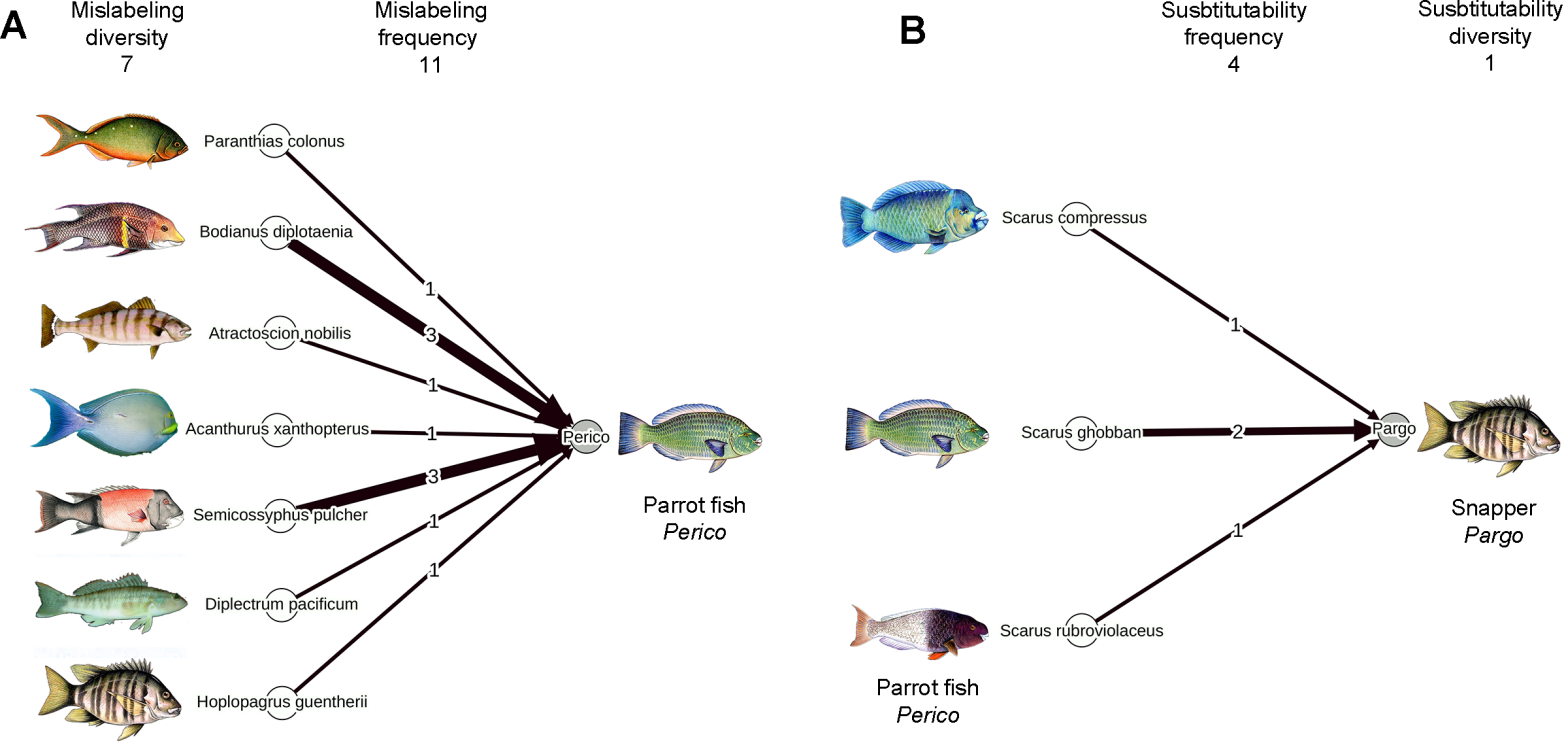
Networks showing results from this study for parrotfish (*perico*) to exemplify the terminology of mislabeling (A) and substitution (B) explained in Table 1 and used throughout the article. The arrows could be read as ”sold as” and describe when other species are mislabeled as the focal species (A) and when the focal species is used as a substitute for other species (B). Images from *A. nobilis* and *D. pacificum* were taken from the Ichthyology Collection at CICIMAR-IPN (http://coleccion.cicimar.ipn.mx/). Image from *A. xanthopterus* was provided by Israel Sanchez Alcantara.

We aimed to map the mislabeling patterns in the city of La Paz, Baja California Sur, Mexico, and find potential explanations that drive these patterns to identify leverage points for future interventions designed to increase the traceability and sustainable management of fish products in La Paz. La Paz, located near the southern tip of the Baja California Peninsula, is the capital of Baja California Sur, a state in Mexico that solely represents ∼21% of the coasts in the entire country and among the 32 states is ranked in the third place in terms of the volume of landed fish and its value (CONAPESCA, 2015). Commercial trade is limited to a single highway that connects the entire Peninsula to mainland Mexico and the USA, in addition to transport via ships and planes. We quantitatively investigated whether the supply of fish species might drive patterns of mislabeling found in fish samples obtained from fish markets and restaurants. In a city like La Paz that is relatively isolated, both geographically and commercially, but with a large and constant supply from local fisheries, the availability of each species based on their local landings could permeate to dictate the availability of each species in fish markets and restaurants, provided that the commercial chain is simple enough and local fisheries represent the main source of fish to the city. However, the availability of each species could be masked at the end of the commercial chain not only by mislabeling in the traditional sense (i.e., when other species are mislabeled as the focal species; Fig. 1A), but could also be confounded when the focal species is used as a substitute for other species; Fig. 1B). We calculated both aspects (mislabeling and use of a species as a substitute) and investigated whether this measure of the net availability of a species in the market was related to local landing data and its relationship with patterns of mislabeling. According to the rationales above, we would expect a higher frequency of mislabeling in species that show low supply and high demand and lower mislabeling in species with high supply and low demand, which we would expect to be used as substitutes for the higher demand species. In addition, we qualitatively explored other potential causes of mislabeling based on our expertise about fish markets in La Paz.

## Materials and Methods

### Sampling

During 2016 and 2017, we obtained samples from 10 distinct fish markets and 30 different restaurants in La Paz, Mexico (total 158 samples). Samples were collected via convenience sampling as part of a citizen science project with the help of 15 volunteers that gathered samples from restaurants and fish markets based on whichever species were available at the sampling location and according to their own preferences and regular fish consumption patterns. Volunteers were instructed to sample only from fish markets and restaurants because we were specifically interested in local small-scale fisheries. Large national or transnational supermarket chains were excluded because they are less likely to sell local products. However, volunteers were not directed to sample any particular species, fish market or restaurant that could have been perceived as preferentially mislabeled or not. Tissue samples were placed in pre-numbered 2 ml tubes topped with 70% ethanol and kept in the fridge (∼8 °C) until processed in the laboratory. For each sample, we collected the following data: date, name of the fish market or restaurant, commercial name or label of the fish species according to the seller, type of sample (fresh, frozen, fried, grilled, breaded, dry), and the price in Mexican pesos paid for the product, either per kilogram (fish markets) or for portion (restaurants).

### DNA extraction, PCR and sequencing

We extracted genomic DNA employing the salting-out method (Miller, Dykes & Polesky, 1988). We amplified via the Polymerase Chain Reaction (PCR) a ∼650 bp fragment of the Cytochrome *c* oxidase I (COI) gene using two pairs of conserved primers and published protocols (Ward et al., 2005). After verification of successful PCR amplification on 1.5% agarose gels stained with RedGel (Biotium), the PCR products were purified and sequenced in the forward and reverse directions using an ABI3730XL DNA analyzer. The resulting sequences were edited by eye using the online software tool BENCHLING (https://benchling.com).

### Establishing mislabeling

To obtain a genetic identification, the edited sequences were compared against two databases: (1) NCBI nucleotide database with the Blast-n search tool (Altschul et al., 1990) using the Megablast algorithm for highly similar sequences; (2) the barcode of life database (BOLD, http://www.barcodinglife.org), against the “species level barcode records”. Species identification corresponded to the top match with sequence similarity of at least 98% present in each database. To establish mislabeling, it is necessary to compare the genetic identification with a list that relates commercial and scientific names (e.g., (U.S. Food and Drug Administration, 2019). Since no such list has been published by official authorities in Mexico, we used an online catalog for fisheries species from the Pacific coast of Mexico (http://catalogo.cicimar.ipn.mx), which details the commercial (common) names for 924 marine species based on three sources: (1) Common names recognized by FAO in Spanish, (2) the Mexico National Fisheries Chart, and (3) common names mentioned in other scientific references (Ramirez-Rodriguez, 2013). We determined mislabeling when the commercial name of a sample did not match in the catalog to any of the commercial names of the species identified with the barcoding analysis. All confidence intervals (CI, *α* = 0.05) around mislabeling rates were calculated using Wilson’s method.

We created a matrix of mislabeling patterns showing the relationship between scientific names identified with the genetic analyses and the commercial name used to sell them. We displayed the matrix in a network using GEPHI (Bastian, Heymann & Jacomy, 2009). To identify the most important nodes, we calculated the weighted degree, *i.e.,* the total number of connections leaving and entering a node weighted by their observed frequency.

### Estimates of net availability and substitutability in the market

To estimate the net availability of a particular species in the market, we took into account both the mislabeling and the substitutability frequency of each species within a network approach as shown in Fig. 1 and explained in detail in Table 1. From the number of samples analyzed under a particular commercial name as told by vendors (Verbal sample number), we first subtracted the number of samples sold as the focal species that were mislabeled according to the genetic analyses as explained above (Mislabeling frequency, Fig. 1A) to obtain the number of samples that were correctly labeled. Then, to the correctly labeled samples, we added the number of samples from the focal species that were used as substitutes for other species (Substitutability frequency, Fig. 1B) to obtain what we called the number of confirmed samples. The number of confirmed samples represents the real number of samples genetically identified for a particular species, after mislabeling and substitution patterns are taken into account, and it was used as a proxy for the net availability of each species in the market.

**Table 1:** Terminology used in the analyses of seafood mislabeling and substitution. For each term, we provide a brief detailed explanation.

**Term**	**Explanation**
Verbal sample number	Number of samples analyzed under the commercial name of the focal species, as communicated by vendor.
Correctly labeled samples	Number of times samples sold as the focal species were correctly labeled.
Mislabeling frequency	Number of times samples sold as the focal species were mislabeled (Fig. 1A).
Mislabeling percentage	Percentage of mislabeled samples relative to the verbal sample number.
Mislabeling diversity	Number of different species sold under the name of the focal species. Used as a proxy for market demand (Fig. 1A).
Substitutability frequency	Number of times samples from the focal species were used as substitutes for other species (Fig. 1B). Used as a proxy for market demand.
Substitutability diversity	The number of different species that the focal species substituted (Fig. 1B). Used as a proxy for market demand.
Confirmed samples	Correctly labeled samples + substitutability frequency. This is the real number of samples genetically identified for the species associated with the commercial name, after considering mislabeling and the use of the species as substitute. Used as a proxy for net availability of a species in the market.
Over/sub-representation	Difference between the verbal sample number and the number of confirmed samples.
Percentage of over/ sub-representation	Percentage of the difference between the verbal sample number and the number of confirmed samples.

We assessed substitutability as a proxy for the demand of a species in the market in three different ways. First, we used substitutability frequency as defined above, under the assumption that species in high demand will be less likely to be used as substitutes for other species, showing lower substitutability frequency. Second, we estimated the number of different species sold under the name of the focal species (Mislabeling diversity, Fig. 1A, Table 1) under the rationale that species with high demand would show higher mislabeling diversity values. Third, we calculated the number of different species that the focal species substituted (Substitutability diversity, Fig. 1B). We expected that species with a high demand, if used as substitutes, would replace only a small number of other species and should show small substitutability diversity values.

### Landings and price estimates from the logbook program

To test the hypotheses that mislabeling is driven by the level of supply of each species and or profits, we employed data from an ongoing small-scale fishery monitoring program led by Sociedad de Historia Natural Niparajá A.C. We obtained data about average monthly and annual landings during 2016–2017 for the 10 main fish species identified in the study. The logbook program is conducted in the region between La Paz and Loreto inside the Gulf of California, a region that represents one of the main sources of fish for the city of La Paz. The program is voluntary and registers fish landings on a daily basis. Fishers or those who receive the production register species, kilograms, fishing location, price per kilogram for the entire fish, and other data. The data are registered daily and have been recorded since 2012. Currently, 70% of the boats in this region participate in the program (NIPARAJA, 2019). For each of the genetically identified species, we obtained information at the start of the commercial chain relating to the average price paid directly to small-scale fishers during 2016–2017, as registered in the logbook program.

Some genetically identified species were not included in the logbook program of small-scale fishers, since they are species either restricted by fisheries authorities to sport fishing activities within 50 nautical miles of the Mexican coasts and their commercialization is prohibited (e.g., bill fishes including marlin and rooster fish) or they are exploited mainly by industrial fisheries (e.g., tuna). We obtained landing data for tuna and marlin from a database that compiles official information about national fisheries landings from the Fisheries Commission in Mexico (dataMares, 2018). We selected the three fisheries offices closest to La Paz that more likely source these species to the local market (Ciudad Constitucion, San Carlos and La Paz) and calculated average annual landings from the three most recent years available in the database. For these two species, we also used the average whole-sale prices published for the city of La Paz, for 2016-2017, found in an online database, maintained by the Secretary of Economy, about monthly prices of various goods and products in Mexico, including the main species of fish (http://www.economia-sniim.gob.mx). We used a linear regression analysis to test the hypothesis that fish landings could help explain the net availability observed for the 10 main commercial species at fish markets and restaurants (confirmed samples, Table 1). We also compared the verbal sample number from each commercial name (Table 1) against fish landings, to test if landings were simply related to the frequency of each commercial name in the study as told by vendors and not specifically to the confirmed samples identified after genetic analyses. Given the large variation in landings between small-scale and industrial fisheries (i.e., tuna), landings in tons were linearized with a log transformation before analyzes.

All the genetically identified species were classified into three price categories. The first class includes large species with an average price >32 MX pesos/kg. The second class are species with an average price between 16-32 MX pesos/kg. The third class are species with an average price between 8-16 MX pesos/kg. These three categories are part of an unofficial price system operated between small-scale fishers and buyers. We defined the three possible scenarios involving mislabeling of a sample: (1) substitution of a species in a lower price category for a commercial name in a higher price category (Up); (2) substitution of a species for a commercial name within the same price category (Same); and (3) substitution of a species in a higher price category for a commercial name in a lower price category (Down). Each category might be associated with distinct causes that might explain the mislabeling patterns at the end of the commercial chain (i.e., at the point of final purchase). For example, we expect that substitution driven by economic gain involves a change from of a species in a lower price category for a commercial name in a higher price category (Up).

### Description of the local value chain

**Figure 2:**
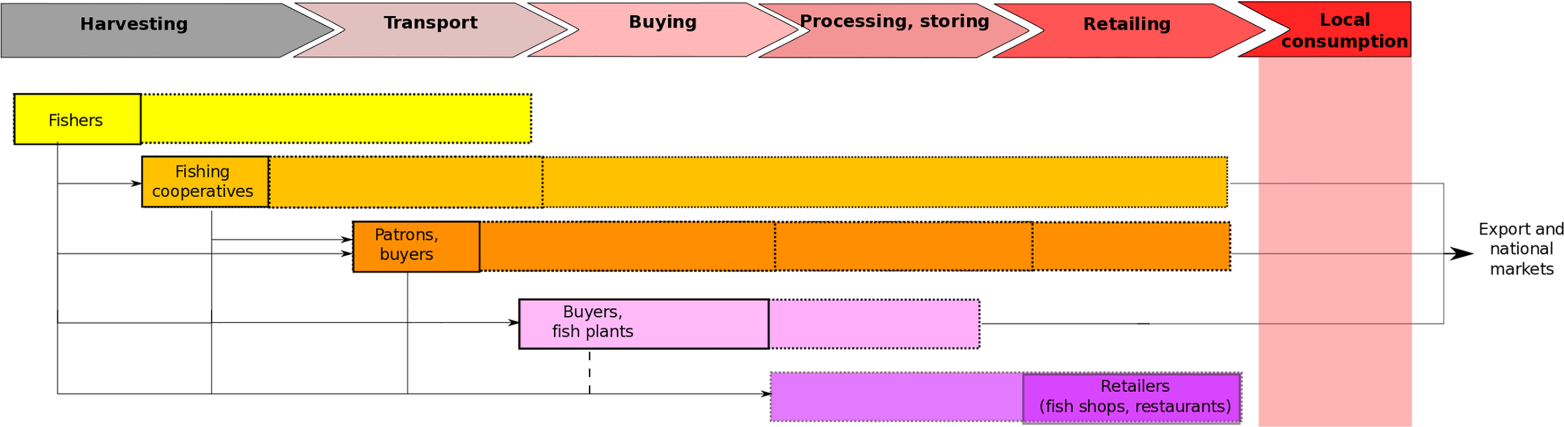
Steps in the local value chain of fish in La Paz, BCS, Mexico, from harvesting by small-scale fishers to local consumption. The different actors are represented by colored boxes, where the continuous line around boxes represents their main role and the dotted lines imply additional roles by some fraction of the actors. Lines connecting actors represent the different potential routes of seafood. Source: Blanca Gonzalez-Mon.

To understand where mislabeling might be happening and the complexity of local seafood trade, we described the value chain that sources fish to La Paz (Fig. 2). Local fish shops and restaurants (retailers) in La Paz mainly source fish from the small-scale fisheries of Baja California Sur, where fishing takes place in both the Gulf of California and the Pacific coast of the Peninsula. Small-scale fisheries fish in 7–9 m motorized vessels (pangas) with 2–3 fishers on board, landing the product at the shore of fishing communities that are often remote (Basurto et al., 2013). This remoteness implies that fish often needs to be transported by road, held on ice in pickups or larger trucks, for 3–10 h (unless fished in the waters adjacent to La Paz). Transport and commercialization may be done by either the fishers themselves (although rarely), by seafood buyers and/or by fishing cooperatives when fishers are organized in cooperative structures (Basurto et al., 2013; González-Mon et al., 2019). Seafood buyers (often called *patrons*) may have diverse informal arrangements with fishers, where for example, fishers exclusively sell to them in exchange for services (e.g., credit) and fishing equipment (Cinti et al., 2010; Basurto et al., 2013). In Mexico, these buyers can also be permit-holders, and thus the ones with legal rights for fishing, and fishers may work under their permit (Basurto et al., 2013; Frawley, Finkbeiner & Crowder, 2019); even if not all patrons establishing informal arrangements are permit-holders (González-Mon et al., 2019). Fishing cooperatives in the region have different degrees of organization, in some cases having a democratic structure and in others functioning more as private enterprises (Frawley, Finkbeiner & Crowder, 2019). Taking into account this diversity of cooperatives, some may commercialize their catch as fishers selling to a seafood buyer (González-Mon et al., 2019), and on the other end, others may develop their own commercialization structure, including even retailing (Fig. 2). Besides these actors that transport seafood from the first point of commercialization, there are also seafood processing plants and buyers that receive the product in the city, who are intermediaries that often sell seafood to tourist, national, or international markets as their main activity (González-Mon et al., 2019). Retailers, whose main activity is to sell fish to local consumers, usually receive fish obtained from the entities/actors mentioned above. As Fig. 2 shows, some actors can perform several activities and functions across the value chain, and even if the structure varies, retailing in this value chain is not generally many steps away from the harvesting process.

## Results

From 158 samples analyzed, we obtained successful PCR products, high-quality consensus sequences and a genetic identification in 124 samples (78.4%). These sequences had an average length of 568 bp (Table S1, GenBank Accessions MT311521–MT311644).

Of the 124 samples included in the final analyses, 74 (60%) were from fish markets and 50 (40%) from restaurants. Both BOLD and GenBank databases produced identical species identification for most samples (113, 91.1%) showing sequence similarity ≥98% (Table S1). For ten samples, there was disagreement between the databases relating to the identification of species within the same genus, but BOLD similarities were comparatively higher (99.8–100%) compared to GenBank (87.1–98%) and thus we followed the BOLD identification for these samples. In one sample, BOLD failed to find a significant match, while GenBank found a 93% match to *Sufflamen fraenatum*, a species of triggerfish from the Indo-Pacific that more likely represented the local *Sufflamen verres*, which seems to be not well characterized in DNA databases.

The 124 samples identified genetically were sold under 19 different commercial names (Table 2) but represented a total of 38 taxonomically distinct species (Table S2). Notably, with the exception of four samples from aquaculture identified as tilapia (*Oreochromis niloticus*) and totoaba (*Totoaba macdonaldi*), and 20 samples identified as yellowfin tuna (*Thunnus albacares*) that could originate from industrial fisheries, imports, or aquaculture, the other 35 species representing 100 samples (80.6%) were locally fished by small-scale fishers according to information from the local logbook program. The 10 most common commercial names had a frequency of 4 to 19 and represented 91.1% of all the samples, while the other 9 commercial names accounted for only 8.9% (Table 2).

**Table 2:** Patterns of mislabeling and substitution for 124 samples sold under 19 commercial names in La Paz, Baja California Sur, Mexico. We include: commercial name (in Spanish), verbal sample number, correctly labeled samples, mislabeling frequency, mislabeling percentage, mislabeling diversity, substitutability frequency, substitutability diversity, confirmed samples, over/sub representation and over/sub representation percentage. The 10 most important commercial names, representing 91.1% of all samples, are shown at the top of the table. See Fig. 1 and Table 1 for detailed descriptions of each term.

#	**Commercialname**	**Verbal sample number**	**Correctly labeled samples**	**Mislabelingfrequency**	**Mislabeling%**	**Mislabelingdiversity**	**Substitutabilityfrequency**	**Substitutabilitydiversity**	**Confirmed samples**	**Over/sub -representation**	**Over/sub representation %**
1	*Jurel*	19	12	7	36.84	7	1	1	13	6	46.15
2	*Cochito*	18	16	2	11.11	1	0	0	16	2	12.5
3	*Atun*	16	15	1	6.25	1	5	4	20	-4	-20
4	*Perico*	16	5	11	68.75	7	4	1	9	7	77.77
5	*Cabrilla*	14	5	9	64.28	4	1	1	6	8	133.33
6	*Pargo*	11	6	5	45.45	4	3	3	9	2	22.22
7	*Pierna*	6	2	4	66.66	4	5	3	7	-1	−14.28
8	*Marlin*	5	3	2	40	1	1	1	4	1	25
9	*Cadernal*	4	3	1	25	1	8	5	11	-7	−63.63
10	*Sierra*	4	0	4	100	4	0	0	0	4	400
11	*Totoaba*	2	2	0	0	0	0	0	2	0	0
12	*Lenguado*	2	1	1	50	1	0	0	1	1	100
13	*Curvina*	1	1	0	0	0	1	1	2	-1	-50
14	*Cazon chico*	1	1	0	0	0	1	1	2	-1	-50
15	*Garropa*	1	0	1	100	1	0	0	0	1	–
16	*Manta*	1	1	0	0	0	0	0	1	0	0
17	*Pez espada*	1	1	0	0	0	0	0	1	0	0
18	*Pez vela*	1	0	1	100	1	0	0	0	1	–
19	*Palometa*	1	1	0	0	0	0	0	1	0	0

The number of species that according to the catalog were associated with each commercial name varied widely (Table S3). For example, while several commercial names are used to refer to a single species (e.g., *cadernal*, *pierna*, *pez espada* and *pez vela*), other commercial names are used for up to 34 species from 5 families and 17 genera (*lenguado*), or 35 species from 4 families and 12 genera (*cabrilla*).

**Figure 3:**
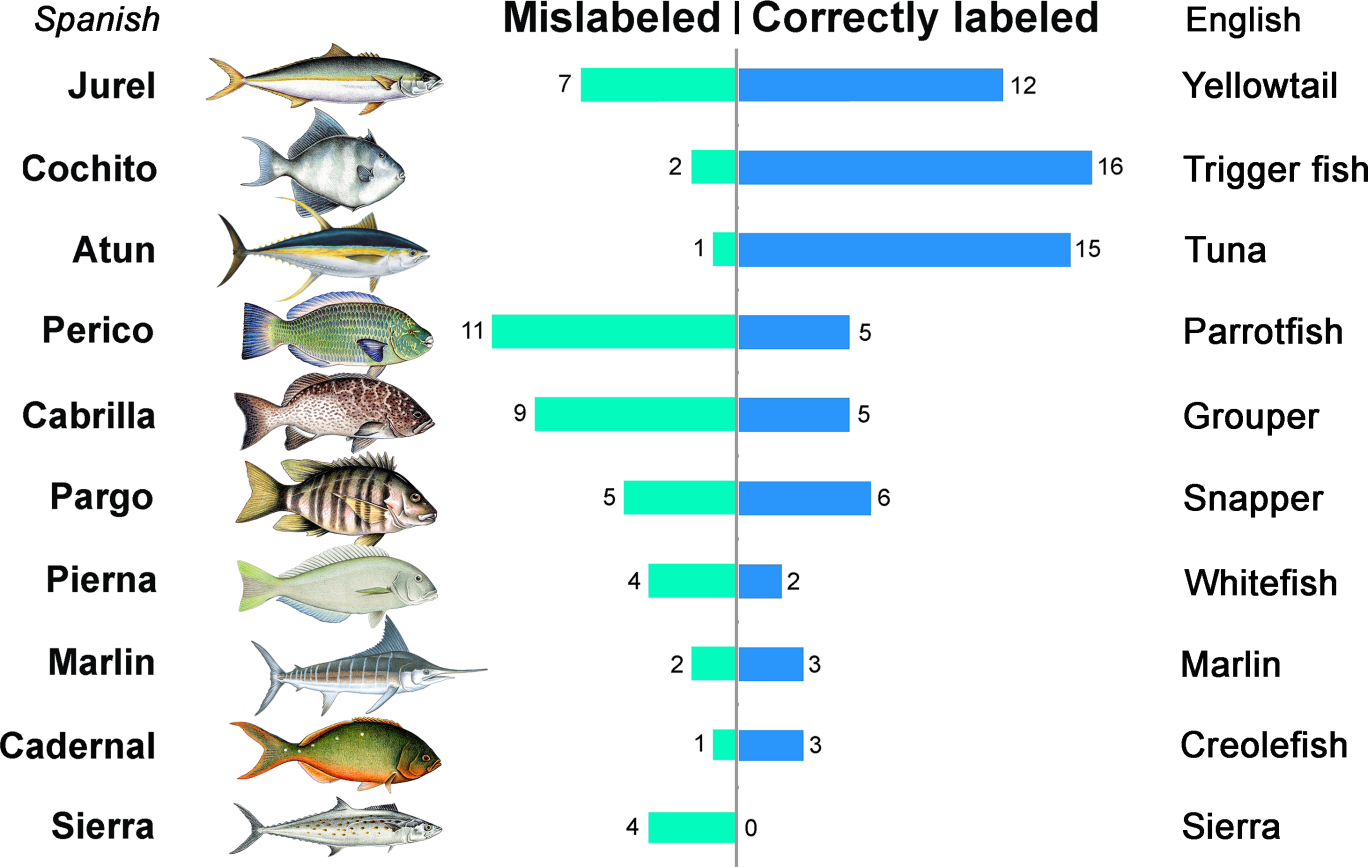
Patterns of fish mislabeling found within the 10 most common commercial names in La Paz, Mexico. Bars show the number of mislabeled and correctly labeled samples and their English translation. The images show species representative for each commercial name.

We found that 49 samples (39.5%, CI [31.4–48.3]) were considered mislabeled according to our criteria. Mislabeling was comparatively lower in fish markets (33.7%, CI [24–45.1) compared with restaurants (48%, C.I. [34.8–61.5]), but the difference was not statistically significant (*X*^2^ = 2.523, *P* = 0.112). Mislabeling rates among the 10 most common commercial names averaged 46.4%, but varied extensively (Fig. 3, Table 2). The most common commercial name (*jurel* or yellowtail) had significant levels of mislabeling (36.84%), while the second and third most common names (*cochito* or triggerfish, and *atun* or tuna) had comparatively lower mislabeling rates (11.11% and 6.25%, respectively). However, all other common commercial names showed higher mislabeling rates, including *perico* (parrot fish, 68.75%), *cabrilla* (grouper, 64.28%), *pargo* (snapper, 45.45%), *pierna* (ocean whitefish, 66.66%), marlin (40%), *cadernal* (Pacific creole fish, 25%), and sierra (100%). Among the 19 common names observed, six common names found at low frequencies (1–2 samples) did not show any mislabeling, including *totoaba*, *curvina* (weakfish), *cazon* (juvenile shark), *manta*, *pez espada* (sword fish) and *palometa* (jack).

With the exception of two species (*cochito* and *sierra*), all the other eight species associated with the 10 most common commercial names were used as substitutes for other commercial names (Substitutability frequency  > 1 in Table 2). This happened at least once for *cabrilla*, *marlin* and *jurel*; three times for *pargo*, four times for *perico*, five times for *atun* and *pierna* and eight times for *cadernal*.

**Figure 4:**
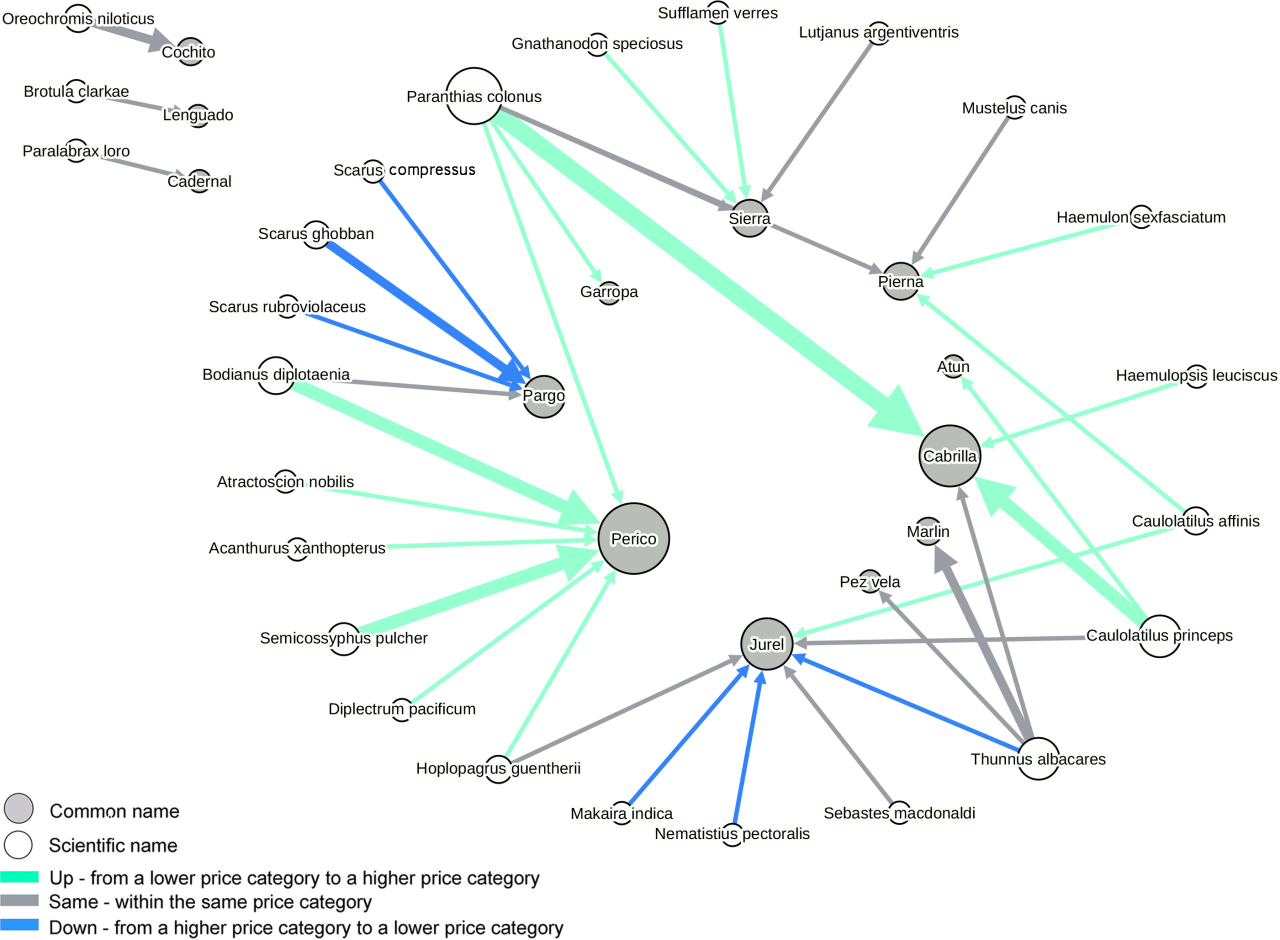
Network showing patterns of 49 instances of mislabeling and substitution found in fish markets and restaurants from La Paz, Mexico. The network connects 13 mislabeled commercial names (grey nodes, in the center of the network) with the 25 species that were used as substitutes (white nodes, in the periphery of the network). Arrow widths represent the frequency of a given mislabeling combination (thickest line = 4 events). The color of arrows represents the three possible scenarios of substitution of species within the three price categories. The size of the circles represents their importance in the network in terms of number and frequency of connections (weighted degree). Note that three species/commercial names on the left are disconnected from everything else.

The number of different species sold under the name of the focal commercial name (Mislabeling diversity in Table 2) varied from only one species (although not the same one) that substituted *cochito*, *atun*, *marlin*, *cadernal*, *lenguado*, *garropa* and *pez vela*, to four species that substituted *cabrilla*, *pargo*, *pierna* and *sierra*, up to seven species that substituted *jurel* and *perico* (Fig. 4). The network displaying the 49 instances of mislabeling found and that connects 13 mislabeled commercial names and 25 species used as substitutes is shown in Fig. 4. With the exception of three commercial names that were substituted by a single species, all the other mislabeling events connecting 10 commercial names and 22 species formed a single network, highlighting the interconnectedness and complexity of mislabeling and substitution patterns. Based on their weighted degree or the number and frequency of connections, the most important commercial name in the mislabeling of fish samples was *perico*, followed in decreasing order of importance by *cabrilla*, *jurel*, *pargo* and *pierna*. The most important species used as a substitute was *Paranthias colonus*, followed in decreasing order of importance by *Thunnus albacares*, *Caulolatilus princeps*, *Bodianus diplotaenia* and *Semicossyphus pulcher.* In terms of the frequency of mislabeling events, those associated with the commercial names *perico* and *cabrilla* were the most frequent (Fig. 4).

Based on our analyses that compared the verbal sample number (i.e., sold as) vs. confirmed samples (i.e., genetic ID) over reported species, for which genetic analyses found fewer samples than told by vendors included (in decreasing order of over-representation, Table 2): *cabrilla,133.33* %, *perico,77.77* %, *jurel,46.15* %, *marlin,25* %, *pargo* 22%, *cochito* 12.5%. Under-reported species, for which genetic analyses identified more samples than originally stated by vendors, included (in decreasing order of under-representation): *cadernal* –63.63%, *atun* –20% and *pierna* –14.28%.

Of the 49 instances of mislabeling found, about half (26, or 53%) included the substitution of a species in a lower price category for a commercial name in a higher price category (Up), 16 (32.6%) were substitutions of a species for a commercial name within the same price category (Same) and only 7 (14.2%) were substitutions of a species in a higher price category for a commercial name in a lower price category (Down) (Fig. 4).

**Figure 5:**
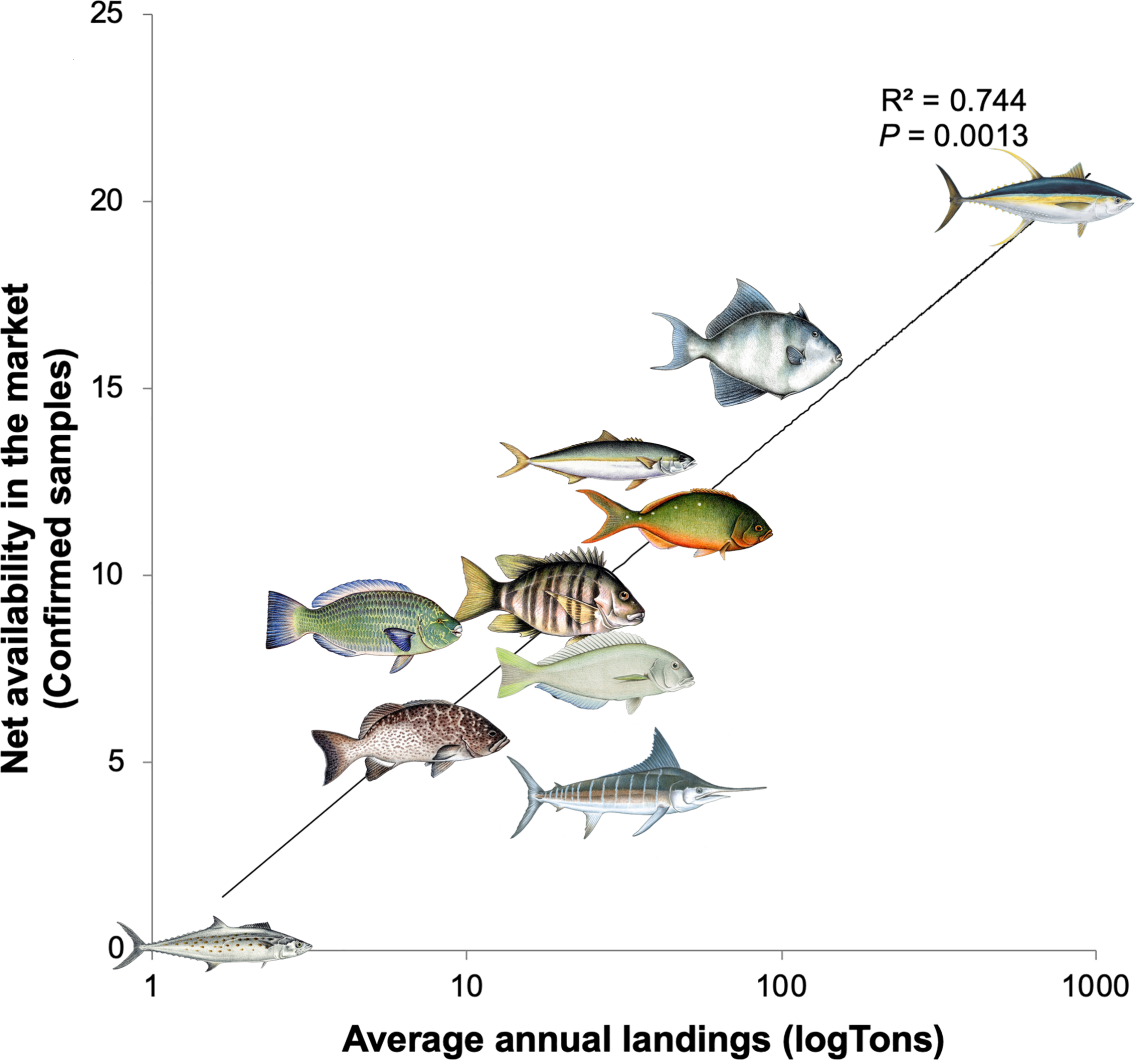
Linear regression analysis showing the relationship of average annual fish landings and net availability of each species in the market for the 10 most frequent commercial names found in La Paz, Mexico. Fish landings as a predictor to the observed net availability in the market (i.e., the total number of genetically confirmed samples, excluding mislabeling and including samples used to substitute other species). The illustrations show species representative for each commercial name shown in Fig. 3.

Linear regression analysis showed the average annual landings for the 10 main commercial species identified were significantly correlated with our measure of net availability observed in the market (Confirmed samples, *R*^2^ = 0.744, *P* = 0.0013, Fig. 5), but not with the verbal sample number (*R*^2^ = 0.112, *P* = 0.3438). Species with higher supply according to local annual landing data (e.g., *atun, cochito*) showed lower mislabeling while species with lower supply (e.g., *perico*, *cabrilla*) were mislabeled more frequently.

## Discussion

Our study analyzed fish mislabeling in the city of La Paz, where 80% of the identified seafood originated from small-scale fisheries in local waters. We focused on two intertwined sides of a complex problem. The first, most commonly studied phenomenon described the frequency of samples that were mislabeled when sold as a particular focal species. The second, often overlooked aspect, focused in the frequency at which the same focal species was used as substitute for other species. We integrated these two processes within a network approach and the outcome provided key insights about the dynamics, complexity and drivers of seafood mislabeling. Local fish landings for the 10 main commercial species that represented 91% of our sample, were highly correlated with our estimate of net availability of species in the market after considering both mislabeling and substitution. This indicates that fish availability at the level of retailers in the city may be similar to fish availability at the first point of commercialization in the fishing communities. The main species recovered from fish markets and restaurants matched all the key species landed by small-scale fisheries in the area and that account for ∼90% of all the landings, according to data from the logbook program. The exceptions include two species within the first-class price category that are exclusively sold to the national and international market (red snapper *Lutjanus peru* or *huachinango*, and star-studded grouper *Hyporthodus niphobles* or *estacuda*) and that were not observed in our study. However, the true availability of the main commercial species was masked at some point of a relatively short commercial chain in the form of over or under representation to the eyes of the final consumer. Below we discuss different factors that might be driving the preferential mislabeling and use of species as substitutes.

Our study documented 40% (CI [31.4–48.3]) mislabeling for fish mainly sourced from artisanal fisheries in fish markets and restaurants in La Paz, Mexico. The rate of mislabeling found was higher than the values recently reported for three other cities within Mexico (range 26.5%–34%) (OCEANA, 2019), and also higher than the average found across 51 studies around the world (30%) (Pardo, Jimenez & Perez-Villarreal, 2016), among 200 studies from 55 countries (19%) (Warner et al., 2016), and compared to the mode from 141 studies in a recent global meta-analysis (24%) (Luque & Donlan, 2019). Although we focused our analyzes in the 10 species with larger sample sizes, the number of samples per species varied widely, and was small for several species. Thus, our results should be taken with caution and verified with larger sample sizes, particularly considering recent studies that have shown under sampling could significantly overestimate true mislabeling rates (Luque & Donlan, 2019). However, obtaining large sample sizes for some commercial products might be difficult because some species seem to be consistently under reported by vendors (e.g., *cadernal*, *pierna*) while they are much frequently sold as substitutes for other species. This observation highlights the need to shift the focus of studies to both how a species is mislabeled and how is also used as a substitute.

One of the most frequent explanations for mislabeling is that seafood substitution is motivated to increase profits. However, economic gain was consistent only with half of the mislabeling events observed, highlighting that mislabeling could be driven by other additional factors acting simultaneously. Among other causes, the supply level of a particular species seems to have at least some influence. We were able to estimate the net availability of species in the market as a proxy for supply by measuring the real number of genetically identified samples of each species, after considering mislabeled samples and those used to substitute other species. Although our results point in the expected direction based on our hypotheses, where mislabeling tends to increase in species in low supply (e.g., *perico*, *cabrilla*), the small sample sizes used to calculate mislabeling rates for some commercial names prevented us from reaching strong conclusions in overall trends. However, further studies could help confirm if measures of net availability in the market match fish landings and explain mislabeling rates in other regions with different species and commercial chains. Additionally, potential issues of multicollinearity that arise from estimating two variables from the same dataset (e.g., mislabeling rates and net availability of species in the market) would need to be addressed.

When there is a high supply of a species, there seems to be very little incentive for their substitution by another species. This was the case for the two species with the highest supply *Thunnus albacares* (*atun*) and *Balistes polylepis* (*cochito*), which showed the lowest levels of mislabeling (6.2% and 11.1%, respectively). In contrast to our results, other recent studies in Los Angeles (USA) (Willette et al., 2017) and across Europe (Pardo et al., 2018) reported high mislabeling rates for yellowfin tuna (78% and 43%, respectively), highlighting strong differences in distinct local contexts. Interestingly, these two species showed contrasting trends regarding their substitutability. While *atun* was a common substitute for other species of large pelagic fishes of similar color and texture, including *jurel*, *pez vela* and *marlin*, *cochito* was never used a substitute. Besides a respectable local reputation as a good tasting and affordable species, the characteristic rhombus body shape of *cochito* make their fillets easily recognizable and harder to disguise.

As the supply level for a species decreases, the incentive to substitute it to meet a continuous demand increases along with mislabeling rates. This seems to be the case for a group of five commercial names (*jurel*, *perico*, *cabrilla*, *pargo* and *sierra*) with the highest levels of mislabeling (ranging from 36.8% to 100%), which were characterized by low to medium levels of supply and medium to high levels of demand in the market based on all our different proxies. These are valued species (1st or 2nd price class) that have a good market acceptance and an established local identity. In this way, these species can act like “vessels” for selling other less popular local species (e.g., *Paranthias colonus*, *Caulolatilus princeps* and *Bodianus diplotaenia*) that are used to meet a demand that seems to exceed the levels of supply at particular times. Based on our simple analysis of three price categories, profit (i.e., “Up” price category) might be an additional reason to use *perico*, *cabrilla* and *sierra* as vessels for lower valued species. These observations are consistent with an effort to maintain the prices and availability of preferred species regardless of their real levels of supply, while avoiding creating new markets for new, unknown species (Crona et al., 2016). Factors affecting the supply of these species in the market could include: (1) the seasonality of the capture of *jurel*, *cabrilla* and *pargo* linked to their reproduction in spawning aggregations (Erisman et al., 2010); (2) the overfishing of species of higher trophic levels, including *cabrilla* and *pargo* (Sala et al., 2004; Calosso et al., 2020); and (3) the preference to sell highly valued species (e.g., *cabrilla*, *pargo*) to higher-value markets such as tourist operators or exporters, which limits their local availability. However, further studies are required to differentiate among the relative contributions of different causes affecting the supply of these species and to understand their influence on the mislabeling patterns identified here, including the confirmation of these trends with larger sample sizes.

Two species in the second-class price category with the third and fourth largest levels of supply according to local landing data, *Paranthias colonus* (*cadernal*) and *Caulolatilus princeps (pierna)*, showed low levels of demand in the market but were frequently used as substitutes for other species, both in terms of frequency and the number of different commercial names they substituted. These two species substituted other similarly valued species, and in some instances, also substituted species of higher value. Both species seem to lack a local identity for their consumption, show a constant increase in landings in recent years (NIPARAJA, 2019), and are common substitutes of other most popular species without consumers noticing. Thus, they show great potential and strong need for improvements in their management and traceability along the commercial chain to avoid overexploitation, for example, through fisheries improvement projects (Bailey et al., 2018).

Interestingly, *jurel* and *pargo*, two species in the second-class category which are available only seasonally, seem to be substituted by higher valued species (i.e., “down” price category), an observation that strongly contradicts the hypothesis of economic gain as the reason behind mislabeling. A different incentive that could influence this mislabeling pattern is selling species that are restricted to sport fisheries within 50 miles from the coast, and that are not allowed to be commercialized in Mexico (DOF 16∕03∕1994 and DOF 25∕11∕2013 ), except in a limited amount when they are caught as up to 10% of bycatch in industrial fisheries. This could be the case of *jurel* used as a vessel for black marlin (*Makaira indica*), roosterfish (*Nematistius pectoralis*) and yellowfin tuna (*Thunnus albacares*). In the case of *pargo*, we observed its use as a vessel to conceal more expensive species of *perico* (*Scarus sp.*) that, although not illegal in the Pacific of Mexico, are the targets of global (Bruno, Cote & Toth, 2019) and local (Gaxiola-Beltran, 2017) campaigns that discourage their consumption given their perceived ecological value. An alternative explanation for the “down” price category is also to sell a perishable first-class product before it spoils, even at a reduced price and under a different name.

It is important to highlight that our study has several additional limitations. First, given that an official list that relates scientific and commercial names was not available for our study (for an example see FDA, 2019), we relied on an extensive catalog that documents the traditional use of commercial names to refer to scientific species. Thus, our estimates of mislabeling rates could be considered conservative, since many commercial names included multiple species that the average person is unlikely to relate to some of the commercial names registered (i.e., species not in bold in Table S3). Second, our price data per kg of the whole fish provides an estimate only in the first step of the value chain. These values can change along subsequent steps of the chain (Fig. 2) before the sale to the final consumer because fish are sold as fillets in fish markets or as part of dishes in a restaurant, and thus their processing yield (proportion of whole fish that is consumed) varies widely between species (some have less flesh) and presentations (some utilize more of the available flesh).

## Conclusions and Recommendations

Our study suggests that actors along a small-scale fisheries value chain (Fig. 2) are able to make some deliberate decisions about what, when and how mislabeling occurs. Although increasing profits are part of the equation, market supply and consumer preferences seem to play an equivalent or even more important role. Availability in the supply level of different species could be affected by their seasonality, overfishing, and local and international demand, while the traditional importance of commercial names seems to be related to perceptions of fish quality. Other factors include national regulations that restrict the fishing or commercialization of certain species and local and global campaigns that discourage specific patterns of consumption. Overall, we suggest hypotheses on the potential causes that explain the mislabeling patterns occurring in a small-scale fisheries value chain. Further research is needed to investigate these causes and potential incentives underlying seafood mislabeling. Understanding these drivers can be key to identifying appropriate interventions that guarantee sustainable seafood harvesting and consumption patterns.

One step to tackle seafood mislabeling in Mexico is to establish an official list of common names linked to scientific names (Reily, 2018), such as the “Seafood list” in the United States (FDA, 2019). Although some studies have suggested that the implementation of monitoring, traceability and labeling programs could help to reduce mislabeling (Ugochukwu et al., 2015; Marin et al., 2018; Willette & Cheng, 2018), our research suggests that while this would help in certain circumstances (e.g., species with high levels of supply) this alone would not solve the issue. Especially since value chain interventions, including certifications that mainly rely on economic incentives, risk not achieving the expected socio-ecological success at the ecosystem level and there is a need to work towards alternative pathways for seafood sustainability (Kourantidou, Kaiser & Blasiak, 2019; Stoll, Bailey & Jonell, 2020). In addition, we suggest interventions that include communication efforts with stakeholders and educational campaigns of consumers (Mariani et al., 2014), including actors along a relatively short value chain (Fig. 2) and retailers (Willette et al., 2018) aimed at decreasing information asymmetry without the full costs and logistics associated with a DNA species authentication program (Ugochukwu et al., 2015). Local interventions could help harness growing interest in the diversity of commercial species available and artisanal food in Mexico and globally to empower consumers to understand and appreciate their local fish and to understand its real availability and seasonality, just like their produce (fruits and vegetables). These changes would imply creating new markets for currently unknown species and could represent an opportunity to improve the retention of profits by small-scale fishers who directly depend on fishing and extract fishing resources that are currently masked.

##  Supplemental Information

10.7717/peerj.10750/supp-1 Supplemental Information 1 Raw data including detailed taxonomic identifications for each of the 124 samples analyzed according to BOLD and GenBank databases For each sample, we show a unique ID, the GenBank accession number assigned, where the sample was purchased, the commercial name, price (MX) per kilogram (fish markets) or per portion (restaurants), if mislabeling was identified or not and the results of the taxonomic identification of the sample according to BOLD and GenBank databases. Samples are presented in the same order in which commercial species are shown in Fig. 3 and Table 2 in the main text.

10.7717/peerj.10750/supp-2 Supplemental Information 2 List of 38 species genetically identified among 124 samples of fish from fish markets and restaurants We show the frequency of each species (N), commercial name in Spanish and common name in English, average beach price during the period 2016-2017, price category (first, second, third class) and price range observed during the same period.

10.7717/peerj.10750/supp-3 Supplemental Information 3 List of the 19 commercial names used to identify the fish species in Spanish (including common English name) and list of associated scientific names Scientific names and taxonomy follow Ramirez-Rodriguez (2013). Species shown in bold are the most common scientific names associated with each commercial name in the city of La Paz, BCS, Mexico, based on expert opinion by the authors. We also include the price category (1st, 2nd or 3rd class) associated to the scientific names (based on average prices shown in Table S2).

## Additional Information and Declarations

### Data Availability

The following information was supplied regarding data availability: All COI DNA sequences described here are available at GenBank: MT311521–MT311644. Raw data regarding sample type, origin and other related information are available in the Supplementary Files.
