# Supplementary material for: Multiple drivers behind mislabeling of fish from artisanal fisheries in La Paz, Mexico
Source: PeerJ. 2021 Jan 29;9:e10750. doi: 10.7717/peerj.10750 (PMC7849509; doi:10.7717/peerj.10750)
Supplement: Supplemental Information 2 — We show the frequency of each species (N), commercial name in Spanish and common name in English, average beach price during the period 2016-2017, price category (first, second, third class) and price range observed during the same period. [file peerj-09-10750-s002.docx]

|  | **Species Found** | **N** | **Commercial name (Spanish)**  **Common name (English)** | **Average price**  **MX (2016-2017)** | | **Price category**** | **Price range**  **MX** |
| --- | --- | --- | --- | --- | --- | --- | --- |
| 1 | *Makaira indica* | 1 | marlin negro (black marlin) | *42** | | First | 37-47 |
| 2 | *Kajikia audax* | 3 | marlin rayado (striped marlin) | *42** | | First | 37-47 |
| 3 | *Nematistius pectoralis* | 1 | pejegallo (roosterfish) | *42** | | First | 37-47 |
| 4 | *Brotula clarkae* | 1 | lengua rosada (Pacific bearded brotula) | *40* | | First | 35-45 |
| 5 | *Scarus ghobban* | 6 | perico barba azul (bluechin parrotfish) | *38* | | First | 30-55 |
| 6 | *Thunnus albacares* | 20 | atún aleta amarilla (yellowfin tuna) | 45* | | First | 40-55 |
| 7 | *Xiphias gladius* | 1 | pez espada (swordfish) | *42** | | First | 37-47 |
| 8 | *Scarus rubroviolaceus* | 2 | perico dátil (bicolor parrotfish) | *38* | | First | 30-55 |
| 9 | *Paralichthys woolmani* | 1 | lenguado huarache (speckled flounder) | 40* | | First | 37-43 |
| 10 | *Mycteroperca rosacea* | 1 | cabrilla sardinera (leopard grouper) | 59* | | First | 55-63 |
| 11 | *Scarus compressus* | 1 | Loro chato (azure parrot fish) | *38* | | First | 30-55 |
| 12 | *Totoaba macdonaldi* | 2 | totoaba (totoaba) |  | | First |  |
| 13 | *Seriola lalandi* | 11 | jurel aleta amarilla, jurel de castilla (yellowtail amberjack) | *20* | | Second | 12-22 |
| 14 | *Dasyatis dipterura* | 1 | raya látigo, mantarraya (whiptail or longtail stingray) | *18* | | Second | 12-18 |
| 15 | *Bodianus diplotaenia* | 4 | vieja mexicana, vieja de fondo (mexican hogfish) | *20* | | Second | 18-24 |
| 16 | *Semicossyphus pulcher* | 3 | vieja californiana, vieja colorada (californian sheephead) | *20* | | Second | 18-24 |
| 17 | *Hoplopagrus guentherii* | 5 | pargo coconaco, pargo mulato (barred snapper) | *28* | | Second | 24-32 |
| 18 | *Lutjanus novemfasciatus* | 2 | Pargo cenizo (dog snapper) | *30* | | Second | 22-32 |
| 19 | *Caulolatilus princeps* | 7 | pierna (ocean whitefish) | *18* | | Second | 12-22 |
| 20 | *Atractoscion nobilis* | 2 | corvina blanca (white weakfish) | *25* | | Second | 18-28 |
| 21 | *Paralabrax auroguttatus* | 1 | cabrilla extranjera, zorrillo (goldspotted sand bass) | *16* | | Second | 14-22 |
| 22 | *Paralabrax loro* | 1 | lucero (parrot sand bass) | *16* | | Second | 14-22 |
| 23 | *Paralabrax nebulifer* | 3 | verdillo (barred sand bass) | *16* | | Second | 14-22 |
| 24 | *Paranthias colonus* | 11 | cadernal (Pacific creolefish) | *18* | | Second | 12-22 |
| 25 | *Mustelus henlei* | 1 | cazón (brown smoothhound) | *20* | | Second | 14-24 |
| 26 | *Mustelus intermedius* | 1 | cazón (brown smoothhound) | *20* | | Second | 14-24 |
| 27 | *Sebastes macdonaldi* | 1 | rocote (mexican rockfish) | *18* | | Second | 12-22 |
| 28 | *Lutjanus argentiventris* | 1 | pargo amarillo (yellow snapper) | 30 | Second | | 22-32 |
| 29 | *Acanthurus xanthopterus* | 1 | cirujano aleta amarilla (yellowfin surgeon fish) | *8* | | Third | 8-12 |
| 30 | *Balistes polylepis* | 16 | cochito (finescale triggerfish) | *9* | | Third | 9-18 |
| 31 | *Haemulon sexfasciatum* | 1 | burro almejero (greybar grunt) | *12* | | Third | 8-12 |
| 32 | *Haemulopsis leuciscus* | 1 | ronco roncacho, roncador (white grunt) | *12* | | Third | 8-12 |
| 33 | *Caulolatilus affinis* | 2 | conejo, guarepa (bighead tilefish) | *12* | | Third | 8-14 |
| 34 | *Sufflamen verres* | 1 | taxi (orangeside triggerfish) | 9 | | Third | 9-18 |
| 35 | *Gnathanodon speciosus* | 3 | palometa (golden jack) | 12 | | Third | 9-18 |
| 36 | *Cirrhitus rivulatus* | 1 | mero chino (giant hawkfish) | 12 | | Third | 9-18 |
| 37 | *Diplectrum maximum* | 1 | babosa, serrano (sand perch) | 12 | | Third | 9-18 |
| 38 | *Oreochromis niloticus* | 2 | tilapia (tilapia) | 15* | | Third | 11-16 |

***** Price from SNIIM

**Sierra and garropa were two commercial names reported, but none of the species found corresponded to these names. We used *Scomberomorus sierra* (Pacific Sierra) from SNIIM, Average price/kg 20 (Second-class) and we used data/price from *M. rosacea* for garropa (First-class) to calculate the shifts in price categories involving these samples.
