# Supplementary material for: Multiple drivers behind mislabeling of fish from artisanal fisheries in La Paz, Mexico
Source: PeerJ. 2021 Jan 29;9:e10750. doi: 10.7717/peerj.10750 (PMC7849509; doi:10.7717/peerj.10750)
Supplement: Supplemental Information 3 — Scientific names and taxonomy follow Ramirez-Rodriguez (2013). Species shown in bold are the most common scientific names associated with each commercial name in the city of La Paz, BCS, Mexico, based on expert opinion by the authors. We also include the price category (1st, 2nd or 3rd class) associated to the scientific names (based on average prices shown in Table S2). [file peerj-09-10750-s003.docx]

| **Commercial name as sold in fish markets and restaurants**  **(Price category)** | **Species corresponding to the commercial name, following Ramirez-Rodriguez (2013)** |
| --- | --- |
| Atún (1st class)  *Tuna* | SCOMBRIDAE:, ***Thunnus albacares*, *T. obesus*, *T. orientalis***, T. *alalunga;* *Euthynnus lineatus*; *Katsuwonus pelamis*. |
| Cabrilla (1st class)  *Grouper* | SERRANIDAE: ***Mycteroperca rosacea*, *M. jordani*, *M. prionura*, *M. xenarcha*; *Paralabrax auroguttatus*, *P. clathratus*, *P. loro*, *P. maculatofasciatus*,** *P. nebulifer*; *Cephalopholis panamensis*; *Alphestes inmaculatus*, *A. mutiguttatus*; *Dematolepis dermatopelis*; *Hyporthodus acanthistius*; *Epinephelus analogus*, *E. itajara*, *E. labriformis*, *E. niphobles*, *E. niveatus*; *Serranus huascarii*, *S. psittacinus*; *Hemanthias peruanus*, *H. signifer*; SCORPAENIDAE: *Sebastes auriculatus*, *S. chlorostictcus*, *S. chrysomelas*, *S. dallii*, *S. godei*, *S. miniatus*, *S. paucispinis*, *S. serranoides*; LOBOTIDAE: *Lobotes pacificus*; TRIGLIDAE: *Bellator xenisma*, *B. gymnostethus*, *B. loxias*. |
| Cadernal (2nd class)  *Pacific creolefish* | SERRANIDAE: ***Paranthias colonus***. |
| Cazón (2nd class)  *Small shark* | **Any juvenile shark.** |
| Cochito (3rd class)  *Trigger fish* | BALISTIDAE: ***Balistes polylepis***, *Pseudobalistes naufragium*, *Sufflamen verres*. |
| Curvina (2nd class)  *Croaker* | SCIAENIDAE: ***Atractoscion nobilis***, *Bairdiella armata*, *B. incista*, *Cheilotrema saturnum*, ***Cynoscion albus*, *C. nannus*, *C. othonopterus*, *C. parvipinnis*, *C. phoxocephalus*, *C. reticulatus*, *C. squamipinnis*, *C. stolzomanni*, *C. xanthulus***, *Isopisthus remifer*, *Larimus acclivis*, *L. argenteus*, *L. effulgens*, *L. pacificus*, *Menticirrhus elongatus*, *M. undulatus*, *Micropogonias altipinnis*, *M. ectenes*, *M. megalops*, *Nebris occidentalis*, *Odontoscion xanthops*, *O. imiceps*, *O. scierus*, *O. strabo*, *O. vermicularis*, *Paralonchurus goodei*, *P. rathbuni*, *Roncador stearnsii*, *Seriphus politus*, *Stellifer chrysoleuca*, *S. illecebrosus*, *Totoaba macdonaldi*, *Umbrina bussingi*, *U. dorsalis*, *U. xanti*, *Pareques fuscovittatus*, *P. viola;* LUTJANIDAE: Lutjanus inermis. |
| Garropa (1st class)  *Big grouper* | SERRANIDAE: ***Mycteroperca jordani*, *M. prionura*, *M. xenarcha*; *Epinephelus exsul*, *E. nigritus***. |
| Jurel (2nd class)  Yellowtail amberjack | CARANGIDAE: ***Seriola lalandi*, *S. rivoliana***; *Uraspis helvola*; *Caranx caballus*, *C. caninus*, *C. lugubris*, *C. melampygus*, *C. orthogrammus*, *C. sexfasciatus*, *C. vinctus*; *Carangoides otrynter*; *Gnathanodon speciosus*; *Chloroscombrus orqueta*; *Decapterus macrosoma*, *D. muroadsi*; *Elagatis bipinnulata*; *Hemicaranx leucurus*, *H. zelotes*; *Oligoplites refulgens*; *Selar crumenophthalmus*; *Selene peruviana*. |
| Lenguado (1st class)  *Solefish* | ACHIRIDAE: *Achirus klunzingeri*, *A. mazatlanus*, *A. scutum*, *Trinectes fonsecensis*; BOTHIDAE: *Bothus constellatus*, *B. leopardinus*; *Engyophrys sanctilaurentia*; *Monolene assaedae*, *M. dubiosa*; PARALICHTHYIDAE: *Ancylopssetta dendritica*; ***Hippoglosina bollmani*, *H. stomata*, *H. tetrophthalmus*; *Paralichthys aestuarius*, *P. californicus*, *P. woolmani*;** *Xystreurys liolepis*; *Citharichthys fragilis*, *C. gilberti*, *C. platophrys*, *C. xanthostigma*, *C. sordidus*; *Cyclopsetta panamensis*, *C. querna*; *Etropus crossotus*, *E. peruvianus*; *Syacium latifrons*, *S. ovale*; PLEURONECTIDAE: *Hypsopsetta guttulata*; *Pleuronichthys ocellatus*, *P. ritteri*; *Parophrys vetulus*; *Microstomus pacificus*, CYNOGLOSSIDAE: *Symphurus malunurus*. |
| Manta (2nd class)  *Manta* | MOBULIDAE: ***Manta birostris*, *Mobula japanica*, *M. munkiana*, *M. tarapacana*, *M. thurstoni***; DASYATIDAE: *Dasyatis dipterura*; GYMNURIDAE: *Gymnura crebripunctata*; MYLIOBATIDAE: *Aetobatus narinari*; *Myliobatis longirostris*; *Rhinoptera steindachneri*; RAJIDAE: *Raja inornata*, *R. rhina*. |
| Marlin (1st class)  *Marlin* | ISTIOPHORIDAE: ***Makaira indica*, *M. mazara*; *Tetrapturus angustirostris*; *Kajikia audax***. |
| Palometa (3rd. class)  *Jack* | CARANGIDAE: *Caranx caballus*, *C. sexfasciatus*, *Carangoides otrynter*, ***Gnathanodon speciosus***, *Hemicaranx leucurus*, *H. zelotes*, *Oligoplites altus*, *O. refulgens*, *Selene brevoortii*, *S. peruviana*, *Trachinotus kennedyi*, *T. paitensis*, *T. rhodopus*,  STROMATEIDAE: *Peprilus medius*, *P. simillimus*, *P. snyderi*, *P. ovatus*; GERREIDAE: *Diapterus aureolus*, *Eugerres lineatus*. |
| Pargo (2nd class)  *Snapper* | LUTJANIDAE: ***Lutjanus aratus*, *L. argentiventris*, *L. colorado*, *L. guttatus*, *L. inermis*, *L. jordani*, *L. novemfasciatus*, *L. peru*, *L. viridis*; *Hoplopagrus guentherii***; SERRANIDAE: *Cephalopholis panamensis*; *Epinephelus niveatus*; *Mycteroperca xenarcha*; MULLIDAE: *Mulloidichthys dentatus*; *Pseudupeneus grandisquamis*; CIRRHITIDAE: *Cirrhitus rivulatus*, HAEMULIDAE: *Conodon nobilis*; GERREIDAE: *Eugerres brevimanus*; SPARIDAE: *Calamus brachysomus*; PRIACANTHIDAE: *Pristigenys serrula*. |
| Perico (1st class)  *Parrotfish* | SCARIDAE: ***Scarus compressus*, *S. ghobban*, *S. perrico*, *S. rubroviolaceus*;** ***Nicholsina denticulata***. |
| Pez espada (1st class)  *Swordfish* | XIPHIIDAE: ***Xiphias gladius***. |
| Pez vela (1st class)  Sailfish | ISTIOPHORIDAE: ***Istiophorus platypterus****.* |
| Pierna (2nd class)  *Ocean whitefish* | MALANCANTHIDAE: ***Caulolatilus princeps***. |
| Sierra (2nd class)  *Sierra* | SCOMBRIDAE: ***Acanthocybium solandri*, *Scomberomorus concolor*, *S. sierra***. |
| Totoaba (1st class)  *Totoaba* | SCIAENIDAE: ***Totoaba macdonaldi***, *Cynoscion xanthulus* |
